# Emerging New Era of Artificial Intelligence and Digital Medicine-directed Management of Chronic Kidney Disease

**DOI:** 10.31662/jmaj.2024-0221

**Published:** 2024-09-20

**Authors:** Kouichi Tamura, Masashi Sakai, Tamio Iwamoto, Shin-ichiro Yoshida, Jin Oshikawa

**Affiliations:** 1 Department of Medical Science and Cardiorenal Medicine, Yokohama City University Graduate School of Medicine, Yokohama, Japan; 2 Department of Nephrology and Hypertension, Yokohama City University Medical Center, Yokohama, Japan; 3 Department of Nephrology, Fujisawa City Hospital, Fujisawa, Japan; 4 Department of Nephrology and Hypertension, Saiseikai Yokohamashi Nanbu Hospital, Yokohama, Japan; 5 Department of Nephrology, JCHO Yokohama Hodogaya Central Hospital, Yokohama, Japan; 6 Department of Nephrology, Yokohama Sakae Kyosai Hospital, Yokohama, Japan

**Keywords:** artificial intelligence, chronic kidney disease, digital medicine, real world data

With the epidemiological transition and aging of the population, heart failure is an epidemic in Japan and worldwide healthcare. It appears that heart failure will become more serious in the near future ^(1), (2)^. Frequently, heart failure coexists with chronic kidney disease (CKD), particularly in Japanese patients ^(3)^. CKD is a clinical diagnostic definition that comprehensively expresses pathological conditions in which kidney damage or decreased kidney function persists ^(4), (5)^. Recent changes in the prevalence of CKD seem to be mainly caused by an increase in Japan’s elderly population ^(6)^. Based on a single-year test in Japanese adults over 20 years of age, CKD cases were estimated to have increased from 13.3 million to 14.8 million between 2005 and 2015 ^(7), (8)^.

Accumulated evidence has shown that CKD increases the risk of cardiovascular diseases (CVD) such as myocardial infarction, stroke, and heart failure, as well as death (cardiorenal linkage). Thus, CKD should be an important target for the primary prevention of CVD ^(3), (9), (10), (11)^. Recent large-scale analysis of a Japanese real-world hospital claims database has shown that the presence of CKD or heart failure in addition to type 2 diabetes significantly increased mortality ^(12), (13)^. In addition, in a large-scale cohort study of CKD patients in Japan, it has been reported that heart failure is more frequent than myocardial infarction and stroke as complications of CKD patients ^(3), (9)^. CKD complications with a risk of acute kidney injury and cardiovascular-kidney comorbidity (i.e., cardiorenal syndrome with heart failure and CKD) are recent pathological features ^(3), (11)^.

Therefore, the Report of the Study Group on Measures Against Kidney Disease, created in close cooperation with academic societies such as the Japanese Society of Nephrology, the Japanese Circulation Society, the Japan Diabetes Society, the Japan Medical Association, local governments, and patient groups, was published by the Health, Labor, and Welfare Association in July 2018 ^(14)^. Based on the report, awareness-raising activities regarding the importance of early detection and treatment of CKD are being carried out ^(14)^.

The Japan Chronic Kidney Disease Database (J-CKD-DB) is a database comprising the electronic health records (EHR) of patients with CKD from university hospitals in Japan ^(15)^. The J-CKD-DB was constructed in 2014 for the longitudinal analysis of medical entities and the clinical course of CKD ^(15)^. The J-CKD-DB is recognized as a nationwide real-world database because of its extensive geographic coverage and diverse patient demographics. It comprises more than 20 university hospitals strategically located across various regions of Japan, ensuring a comprehensive geographic representation reflective of the national population of patients with CKD. Furthermore, the database includes a broad spectrum of CKD stages, comorbidities, and demographic characteristics, making it representative of the Japanese population. The advantages of real-world database-based research over randomized controlled trials include larger sample sizes and longer follow-up periods ^(16), (17), (18)^.

Recently, clinical application of ICT/IoT technologies has started in treatment. The American Diabetes Association has been actively promoting ICT/IoT use because diabetes treatment is highly compatible with wearable devices such as insulin pumps and continuous glucose monitors. (https://www.diabetes.org/healthyliving/devices-technology). Although there are issues related to security, privacy, and data ownership, ICT/IoT-based therapies are called digital therapeutics, and they are attracting attention as an essential means to ensure healthcare access for the growing number of diabetes patients, to provide high-quality healthcare, and to control the progression of complications ^(19)^.

The pandemic of novel coronavirus disease (COVID-19) has strained healthcare resources, and there is concern that hospital visits and face-to-face care increase the risk of viral transmission. Diabetic patients are particularly at risk for severe COVID-19 infection, and a combination of factors from urban lockdown due to the pandemic, interruption of medical visits due to refraining from going out, lifestyle changes, decreased physical activity, and stress may lead to more severe complications, including diabetic kidney disease through poor glycemic control ^(20), (21), (22)^. In this context, there are international proposals to utilize ICT and IoT in conventional medicine ^(23)^, and digital therapeutics is gaining momentum. The Japan Diabetes Society also formulated the 4th Five-Year Strategic Plan Against Diabetes, which reports the importance of promoting self-management using ICT and IoT and using personal health records (PHR) as countermeasures against the threat and pandemic of emerging and re-emerging infectious diseases (http://www.jds.or.jp/modules/education/index.php?content_id=118) ^(24)^. The prolonged pandemic of COVID-19 has reaffirmed the importance of ICT and IoT in Japan and abroad, and the use of ICT and IoT in diabetes care is more active than ever ^(25)^. The prolonged pandemic of COVID-19 has reaffirmed the importance of ICT/IoT in Japan and abroad.

A recent study evaluated the effectiveness of mobile health (mHealth) intervention for diabetic kidney disease patients by conducting a 12-month randomized controlled trial among 126 patients with type 2 diabetes mellitus, with moderately increased albuminuria recruited from 8 clinical sites in Japan ^(26)^. The study showed that for the first time, a lifestyle intervention via mHealth achieved a clinically significant improvement in moderately increased albuminuria ^(27)^.

Deep learning is a method in artificial intelligence (AI) that teaches computers to process data in a way inspired by the human brain. With the development of ICT/IoT/EHR/PHR strategy, further translational study is warranted to evaluate whether deep learning techniques demonstrate superior accuracy in the exact diagnosis of the etiology of CKD and estimation of future kidney function compared to conventional statistical methods. In this issue of JMA Journal, Kanda comprehensively reviewed the recent progress in the development of AI systems employing clinical and pathological information in the emerging new era of digital medicine (ICT, IoT, EHR, and PHR)-directed management of CKD ^(28)^.

## Article Information

### Conflicts of Interest

None

### Disclaimer

Kouichi Tamura is one of the Editors of JMA Journal and on the journal’s Editorial Staff. He was not involved in the editorial evaluation or decision to accept this article for publication at all.
